# Hemoadsorption as Adjuvant Therapy in Acute Respiratory Distress Syndrome (ARDS): A Systematic Review and Meta-Analysis

**DOI:** 10.3390/biomedicines11113068

**Published:** 2023-11-16

**Authors:** Csenge Erzsébet Szigetváry, Caner Turan, Emőke Henrietta Kovács, Tamás Kói, Marie Anne Engh, Péter Hegyi, Gábor Csukly, Zoltán Ruszkai, Zsolt Molnár

**Affiliations:** 1 Department of Anesthesiology and Intensive Therapy, Semmelweis University, 1085 Budapest, Hungary; 2 Centre for Translational Medicine, Semmelweis University, 1085 Budapest, Hungary; 3 Department of Stochastics, Institute of Mathematics, Budapest University of Technology and Economics, 1111 Budapest, Hungary; 4 Institute for Translational Medicine, Medical School, University of Pécs, 7624 Pécs, Hungary; 5 Institute of Pancreatic Diseases, Semmelweis University, 1083 Budapest, Hungary; 6 Department of Psychiatry and Psychotherapy, Semmelweis University, 1085 Budapest, Hungary; 7 Department of Anaesthesiology and Intensive Therapy, Pest County Flór Ferenc Hospital, 2143 Kistarcsa, Hungary; 8 Department of Anesthesiology and Intensive Therapy, Poznan University, 60-806 Poznan, Poland

**Keywords:** hemoadsorption, blood purification, cytokine removal, ARDS, acute lung injury, cytokine adsorption, cytokine storm, COVID-19

## Abstract

Background: Acute respiratory distress syndrome (ARDS) is often a consequence of a dysregulated immune response; therefore, immunomodulation by extracorporeal cytokine removal has been increasingly used as an adjuvant therapy, but convincing data are still missing. The aim of this study was to investigate the effects of adjunctive hemoadsorption (HA) on clinical and laboratory outcomes in patients with ARDS. Methods: We performed a systematic literature search in PubMed, Embase, CENTRAL, Scopus, and Web of Science (PROSPERO: CRD42022292176). The population was patients receiving HA therapy for ARDS. The primary outcome was the change in PaO2/FiO2 before and after HA therapy. Secondary outcomes included the before and after values for C-reactive protein (CRP), lactate, interleukin-6 (IL-6), and norepinephrine (NE) doses. Results: We included 26 publications, with 243 patients (198 undergoing HA therapy and 45 controls). There was a significant improvement in PaO2/FiO2 ratio following HA therapy (MD = 68.93 [95%-CI: 28.79 to 109.06] mmHg, *p* = 0.005) and a reduction in CRP levels (MD = −45.02 [95%-CI: −82.64; −7.39] mg/dL, *p* = 0.026) and NE dose (MD = −0.24 [95%-CI: −0.44 to −0.04] μg/kg/min, *p* = 0.028). Conclusions: Based on our findings, HA resulted in a significant improvement in oxygenation and a reduction in NE dose and CRP levels in patients treated with ARDS. Properly designed RCTs are still needed.

## 1. Introduction

Acute respiratory distress syndrome (ARDS) remains one of the greatest challenges in critical care and is associated with high mortality [1,2,3]. Since its first description in 1967 [4], until the most recent Berlin criteria [3] established in 2012, several efforts have been attempted to reach a consensus on its appropriate definition [3,5]. However, ARDS is not a definitive disease [6]; therefore, there is no specific therapy for its treatment other than supportive therapy, including mechanical ventilation, paving the way to gain time to find and treat the initial or underlying decompensating factors. Mechanical ventilation remains the cornerstone of ARDS management, which has considerably improved over recent years [7,8]. Nevertheless, ventilator-induced lung injury still represents a major concern, and reducing the time spent on mechanical ventilation is of the utmost importance [1].

The complex pathology of ARDS often results from a dysregulated inflammatory response (of infectious or non-infectious etiology) characterized by an excessive release of circulating pro-inflammatory and anti-inflammatory mediators, leading to a condition known as a “cytokine release syndrome” [9]. In the fields of septic shock and cardiac surgery, extracorporeal blood purification by hemoadsorption (HA) has recently been introduced into clinical practice as an adjunctive therapeutic approach to immunomodulation [10]. Because of the common association between ARDS and hyperinflammatory conditions, immunomodulation applying HA seems to be justified in this setting too [10].

Designed for hemoadsorption, HA columns are commonly used as pre- or post-filter during continuous renal replacement therapy (CRRT), incorporated into a bypass of an extracorporeal membrane oxygenation (ECMO) circuit or applied as a stand-alone device. Adsorption can be performed by either special hemofilters or adsorption columns. Hemodiafilters provide renal support by transmembrane convection with diffusion, whereas adsorbing cytokines and toxins as well [11]. Adsorption columns are usually size-selective columns (e.g., containing hydrophobic, styrene−divinylbenzene beads with a diameter of 300–800 µm [12]). These biocompatible polymer beads are highly porous, with pore diameters ranging from 0.8–50 nm [13].

Despite the increasing number of publications over the past eight years on the pathophysiological rationale and the use of hemoadsorption in severe acute respiratory failure, there is still a lack of solid clinical evidence to define the role of this treatment modality in general. Moreover, the immediate effects of hemoadsorption on clinical and laboratory measures often remain uncertain [14,15]. Therefore, this systematic review and meta-analysis aims to summarize the currently available data on hemoadsorption in patients treated for severe ARDS.

## 2. Materials and Methods

Our systematic review was registered in advance in PROSPERO (CRD42022292176). This research was conducted following the recommendations of the Cochrane Handbook for Systematic Reviews of Interventions Version 6.1 [16]. We reported the results according to the Preferred Reporting Items for Systematic Reviews and Meta-Analyses (PRISMA) 2020 Statement [17].

### 2.1. Eligibility Criteria

We included prospective and retrospective studies, case reports, and case series in which adult patients with ARDS received hemoadsorption therapy (intervention group) with or without comparing them with patients on standard medical treatments, as defined by current guidelines (control group). We used the Berlin criteria to define ARDS [3].

### 2.2. Primary and Secondary Outcomes

Our primary outcome was the change in oxygenation, as indicated by the PaO2/FiO2 ratio before and after HA. Secondary outcomes included inflammatory response before and after HA, as indicated by C-reactive protein (CRP), procalcitonin (PCT), interleukin levels, and vasopressor support. For safety outcomes, we collected data on white blood cell count, red blood cell count, hemoglobin, platelet count, neutrophil count, and serum albumin levels. We also collected data on 28-day ventilator-free days, changes in respiratory mechanics (static and dynamic compliance, plateau, and driving pressure), vasopressor-free days, CRRT/RRT free days, 28-day organ support-free days, need for rescue therapy (prone and ECMO) readmission rate, length of stay in ICU and hospital, and mortality.

### 2.3. Search Methods

We performed a systematic search in five databases (MEDLINE via PubMed, Embase, Cochrane Central Register of Controlled Trials (CENTRAL), Scopus, and Web of Science) on 17 December 2021, using a predefined search query, and the search was repeated on 25 February 2023. No filters or restrictions were applied.

### 2.4. Selection of Studies Included and Data Extraction

The records were collected using a citation manager (Endnote X9, Clarivate). After duplicate removal, two independent review authors (C.E.S. and C.T.) screened the records according to predefined eligibility criteria, by title and abstract, and, later, full text. Discrepancies were resolved in both phases by a third review author (F.D.). Cohen’s kappa coefficient (κ) was calculated for both selection stages. κ values ≤ 0 were interpreted as no agreement, 0.01–0.20 as none to slight agreement, 0.21–0.40, as fair agreement, 0.41–0.60 as moderate agreement, 0.61–0.80 as substantial agreement, 0.81–1.00 as almost perfect agreement, and 1.00 as perfect agreement.

### 2.5. Risk of Bias and Certainty of Evidence Assessment of the Included Studies

Following the recommendations of the Cochrane Collaboration, we investigated the risk of bias for all of the studies included. Two investigators (C.E.S. and C.T.) independently assessed the quality of the studies. We utilized the Critical Appraisal Tool of the Joanna-Briggs Institute for case reports and case series. For cohort studies, we used the ROBINS-I and, finally, for RCTs, the RoB-2 tool [18,19,20]. If the domains in a given study were all low risk, the overall assessment had a low risk of bias. If at least one domain was high risk, or at least three ‘some concerns’ were included, then the overall assessment was ‘high risk’ of bias. All of the other cases were rated as ‘some concerns’. Any disagreements were solved by a third author (E.H.K.). The quality of the studies included was assessed using the GRADE-Pro program, based on the recommendations of the Cochrane Collaboration [21]. Disagreements were resolved by a third review author (E.H.K.).

### 2.6. Data Synthesis and Statistical Analysis

Statistical analyses were performed using the statistical software R (version 4.1.2.) [22]. The meta-analysis followed the advice of Harrer et al. [23]. For each continuous outcome, we meta-analyzed before- and after-treatment means and mean differences. For one outcome, the difference between the before- and after-treatment means was also available in a control group in at least one study. In this case, we also meta-analyzed the difference in before- and after-treatment mean differences.

We used the classical inverse variance method with the restricted maximum likelihood estimator. As only a few studies contributed to the meta-analysis, the Hartung-Knapp adjustment was applied [24]. Besides the prediction interval, heterogeneity was assessed by calculating the I^2^ measure and its confidence interval and by performing the Cochrane Q test. I^2^ values of 25%, 50%, and 75% were considered as low, moderate, and high heterogeneity, respectively.

In a few cases, only the median and interquartile range of the continuous outcome were available. In these cases, we used the default method of the metacont() R function; we estimated the mean and the standard deviation using the methods described by Luo et al. and Wan et al., respectively [25,26].

Although standard deviations of the outcome before and after the treatment were available or could be estimated in all cases, the standard deviation of the change was missing. Following the instructions of the Cochrane Handbook [16], we inputted several different correlations from the range [−0.5, 0.9]. All of the employed correlations produced similar results. Published results were produced using the inputted correlation of 0.8. We also included the zero correlation results as a Statistical Supplementary Material. We highlight that the studies’ confidence intervals are more significant in the latter case.

From the meta-analyses described above, we excluded case reports or case series in which very few eligible patients were observed. These excluded results were visualized on boxplots, and a Wilcoxon test was performed to see whether the magnitude of the before and after values differed.

## 3. Results

**Figure 1:**
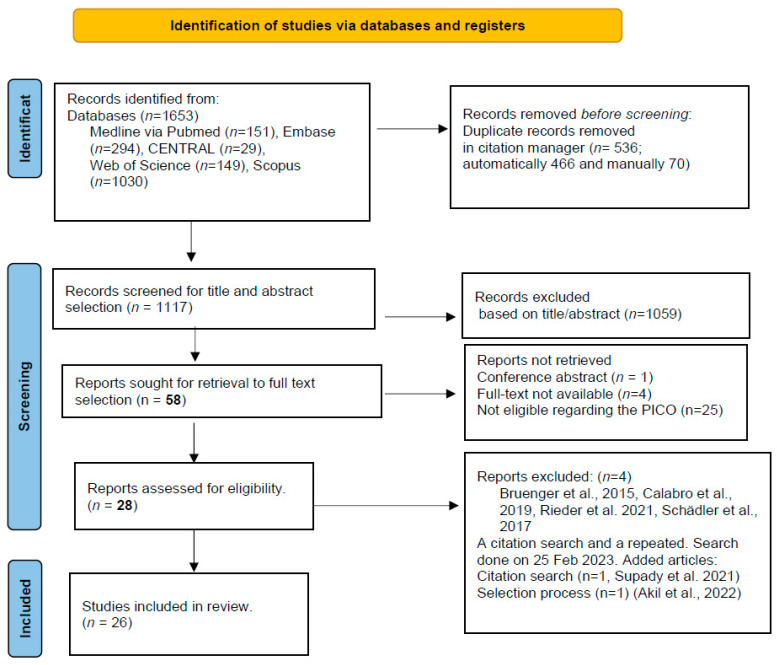
PRISMA 2020 flow chart. Steps of the search and selection process of the included articles [46,50,51,52,53,54].

Our systematic search resulted in 1653 records. After duplicate removal, 1117 articles went through title and abstract selection, during which we identified 58 articles. After full-text selection, 28 met the eligibility criteria. We achieved substantial agreement for both selection phases (κ = 0.65 and κ = 0.67). During further data extraction, four articles were found to be ineligible, and we included two more articles based on a search using references from studies already included based on a second systematic search of the databases to update our selection. Finally, we identified 26 publications (12 case reports [27,28,29,30,31,32,33,34,35,36,37,38], 9 case series [14,15,39,40,41,42,43,44,45], 3 retrospective studies [46,47,48], and 2 randomized controlled trials [49,50]) for quantitative synthesis, with a total number of 243 patients (198 undergoing HA therapy and 45 controls). The process of article selection is depicted in Figure 1. The included articles, studies, and their characteristics are summarized in Table 1.

### 3.1. Devices Used

The vast majority of patients (*n* = 23) received HA therapy using a CytoSorb^®^ device (Monmouth Junction, NJ, USA). Two publications used the oXiris AN69ST/PEI (oXiris^®^, Baxter^®^, Meyzieu, France) [31,55] and the other articles used HA-380 or HA-330 cartridges (Jafron Biomedical Co., Ltd., Zhuhai, China) [29,49].

### 3.2. Primary Outcomes

**Figure 2:**
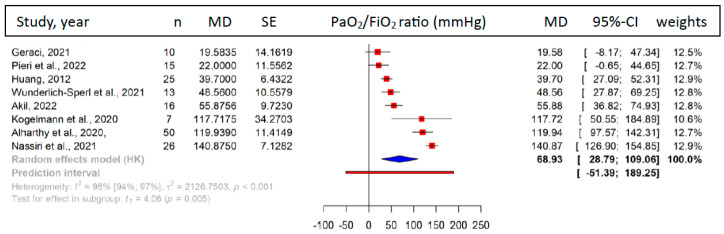
PaO2/FiO2 ratio. Forest plot of the mean difference in PaO2/FiO2 ratio, showing a significant improvement after hemoadsorption treatment (MD = 68.93 mmHg, *p* = 0.005) [14,39,40,42,43,47,48,49].

Pooled data from eight publications (patient number, *n* = 162) reporting on our primary outcome showed a significant improvement in the PaO2/FiO2 ratio after HA therapy (MD = 68.93 [95%-CI: 28.79 to 109.06] mmHg, I^2^ = 96%, *p* = 0.005) (Figure 2). Data pooled from seven case reports of individual patients (*n* = 7) showed a tendency for improvement, although not reaching statistical significance (*p* = 0.15, Figure S1).

### 3.3. Secondary Outcomes

#### 3.3.1. Inflammatory Biomarkers

**Figure 3:**
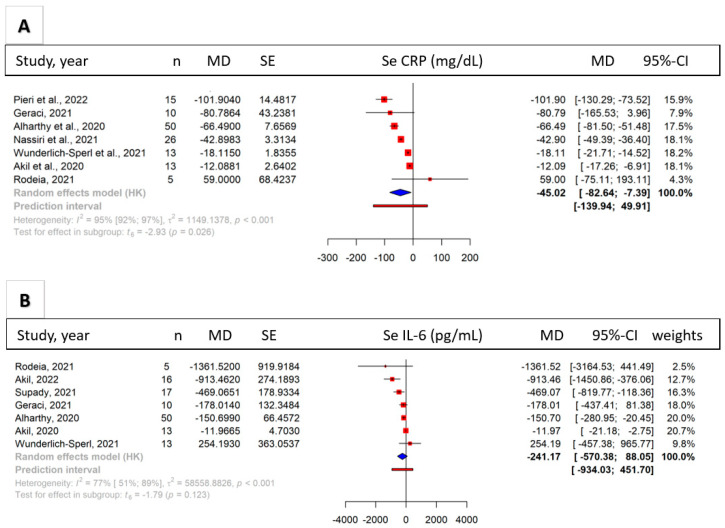
Serum CRP and IL-6. Forest plots of the difference of mean differences of Se CRP (**A**) and Se IL-6 (**B**) after hemoadsorption treatment. The Se CRP was significantly lower (*p* = 0.045) whereas the IL-6 showed a non-significant of reduction after the hemoadsorption therapy (*p* = 0.123) [14,39,42,43,45,46,47,48,50].

Seven publications (*n* = 132) reporting data of COVID-19 patients showed a significant reduction in serum CRP levels after treatment (MD = −45.02 [95%-CI: −82.64; −7.39] mg/dL, I^2^ = 95%, *p* = 0.026) (Figure 3A). The results shown in the Forest plot were also supported by the pooled individual cases, as shown in Figure S2 (*p* = 0.008). Serum IL-6 (interleukin 6) levels were reported in seven publications (*n* = 124). The data revealed a tendency of decreasing serum IL-6 levels after HA therapy (MD = −241.17 [95%-CI: −570.38 to 88.05] pg/mL, I^2^ = 77%, *p* = 0.123) (Figure 3B). These findings were also supported by pooled data from individual cases (*p* = 0.016, Figure S3).

#### 3.3.2. Effects on NE and Lactate

**Figure 4:**
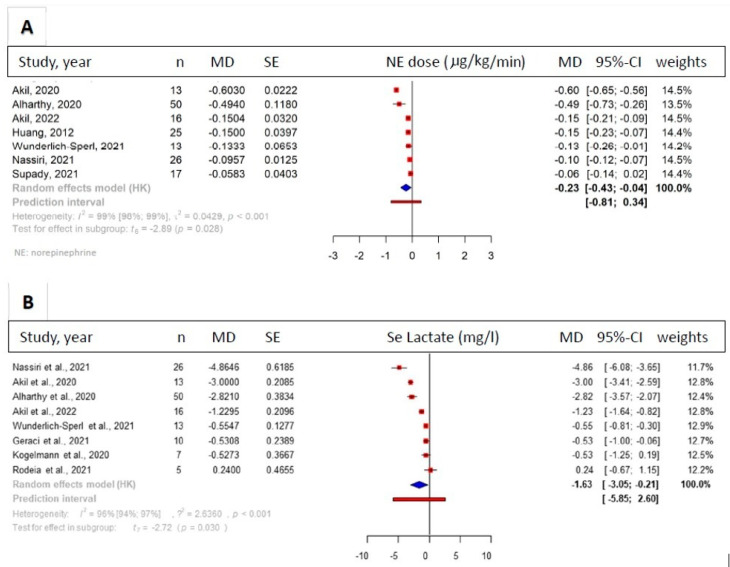
Norepinephrine (NE) doses and serum lactate level. Forest plots of change in required dose of NE (**A**) and the level of rerum lactate (**B**) after hemoadsorption (HA) treatment. After hemadsorption, the required dose of NE was significantly lower (MD = −0.23 μg/kg/min, *p* = 0.028) and serum lactate also showed a significant reduction (MD = −1.63 mg/L, *p* = 0.030) after HA therapy [14,39,40,42,43,45,46,47,49,50].

We analyzed data from seven studies (*n* = 160) reporting the changes in NE before and after HA. The results showed a significant reduction in NE requirement after HA therapy (MD = −0.23 [95%-CI: −0.43 to −0.04] μg/kg/min, I^2^ = 99%, *p* = 0.028) (Figure 4A). An analysis of the data from four studies [46,47,49,50] (*n* = 126) also including a control group showed a tendency toward reduction in vasopressor requirement in patients receiving HA therapy, compared with standard medical treatment. In this case, we found a tendency for decreased NE requirement in the HA group (MD = −0.12 [95%-CI: −0.29 to 0.05] μg/kg/min, I^2^ = 74%, *p* = 0.108); however, the results were not significant, and the certainty of evidence was graded as ‘low’ (Figure S4, Table S1). Serum lactate levels showed a significant decrease after HA therapy, (MD = −1.63 [95% CI: −3.05 to 0.21] mg/L, I2 = 96%, *p* = 0.030) (Figure 4B).

#### 3.3.3. Other Laboratory Findings and Safety Outcomes

The reduction in PLT count did not reach statistical significance in three publications (*n* = 73) reporting data on COVID-19 patients (MD = −57.12 [95%-CI: 181.14 to 66.90] G/L, I2 = 92%, *p* = 0.186) (Figure S5A). We did not find any significant effect of HA therapy on WBC (white blood cell) count when analyzed using individual data (*p* = 0.58, Figure S5B).

No device-related adverse events were reported, irrespective of the platform used.

#### 3.3.4. Length of Stay and Mortality

On the basis of the data extracted from three studies (*n* = 92) comparing HA therapy to standard medical treatment, we found no difference in the length of ICU stay (MD = 1.17 [95%-CI: −18.61 to 20.96] days, I^2^ = 64%, *p* = 0.82) among patients with a ‘low’ certainty of evidence (Figure S6 and Table S1). Five studies reported data on mortality, but assessment varied between 28 to 90 days. We found a non-significant reduction in mortality (RR = 0.64 [95%-CI: 0.11 to 3.65], I^2^ = 80%, *p* = 0.52) in patients receiving HA therapy with a ‘very low’ certainty of evidence (Figure S7 and Table S1).

#### 3.3.5. Subgroup Analysis COVID-19 and Survival

We performed a subgroup analysis on the COVID-19 population. By removing non-COVID cases, only PaO2/FiO2 ratio differences remained significant (Figure S8A–D). In addition, due to a lack of data, it was not possible to analyze and compare survivals and non-survivals (Figure S8A–D).

### 3.4. Outcomes with Insufficient Reporting

As they were underreported in the included studies, we did not find sufficient data on further laboratory findings. The outcomes for any aspects of mechanical ventilation (duration and respiratory parameters) or organ support therapies (vasopressor-free days, CRRT/RRT free days, and 28-day organ-support-free days) were also underreported to be included in any analysis. We were also unable to perform subgroup analyses based on ECMO therapy or prone positioning.

### 3.5. Risk of Bias Assessment and Quality Assessment of the Studies Included

We used the Cochrane Collaboration Risk of Bias tool as part of the quality assessment. Any disagreements were solved by a third author (E.H.K.). The risk of bias and the summary table of findings of the quality assessment (GRADE) of the included studies can be found in the supplementary material (Table S1 and Figure S9A–D).

## 4. Discussion

Hemoadsorption as an adjunctive therapy for different critical conditions associated with hyperinflammation has received increasing interest over the last 10 years. One potential indication is ARDS; however, a comprehensive analysis of the available results has not yet been performed. Our systematic review, which is the first in this field, revealed that only few studies on HA in ARDS have been performed, and high-quality data were missing, but we detected a significant signal toward improved oxygenation, attenuation of inflammatory response, and reduced vasopressor requirements after HA application, without any serious adverse events reported.

### 4.1. Hemoadsorption as Adjuvant Therapy in ARDS

The activation of the innate immune system leads to pro-inflammatory signaling and the release of an array of cytokines [56] and chemokines, as well as activation of alveolar macrophages, resulting in endothelial and alveolar injury [57]. Undoubtedly, most ARDSs result from the uncontrolled release of inflammatory mediators that contribute to severe lung injury. The hypothesis that a predominant systemic inflammation results from a loss of control over the host’s immune response provides the theoretical rationale for extracorporeal cytokine removal as an adjunctive therapy in certain hyperinflammatory conditions, including ARDS. Indeed, our results confirm the substantial research activity in this field, and hemoadsorption is increasingly used in patients who develop severe hypoxemic respiratory failure.

Among the included publications, the least commonly applied was the oXiris filter, followed by Jafron’s HA-330 and HA-380, and the most investigated hemoadsorber was the CytoSorb^®^.

In brief, the oXiris filter (oXiris^®^, Baxter^®^, Meyzieu, France) has an additional polycationic polyethyleneimine (PEI) layer, with a further adsorptive capacity, complemented by a thin layer of biologically inactive heparin [11]. The adsorption mechanism seems to involve ionic and hydrophobic bindings. A prospective, multicenter trial evaluating the AN69ST membrane further confirmed these findings for TNF-α (tumor necrosis factor-α), IL-1β, IL-6, IL-8, IL-10, and HMGB-1 removal [58]. We were able to identify only two publications in our systematic search in which the oXiris filter was applied. Both found a reduction in inflammatory marker levels in their COVID-19 patient population [31,55].

The HA-330 and HA-380 cartridges (Jafron Bio-medical Co., Guangdong, China) are dedicated hemoadsorption columns. The device adsorbs substances in a size-selective manner, but specific literature on the mode of action remains scarce. Two small RCTs on sepsis-related acute lung injury and sepsis described an effective reduction in cytokine levels and an improvement in hemodynamics and mortality [49,59].

Undoubtedly, the most thoroughly investigated hemoadsorption device is CytoSorb (CytoSorbents, Monmouth Junction, NJ, USA), with treatments approaching 200,000 worldwide. It is a CE mark-approved, whole-blood-based adsorptive extracorporeal blood purification technology, designed to effectively adsorb and eliminate elevated levels of cytokines and other inflammatory mediators up to a molecular weight of around 60 kDa from the blood. The adsorbent bead resin consists of polystyrene divinylbenzene coated with a biocompatible polyvinylpyrrolidone, which effectively adsorbs smaller molecules via direct retention through different pore sizes, as well as hydrophobic interactions, electrostatic attraction, hydrogen bonding, and van der Waals forces [60].

### 4.2. Modulating the Inflammatory Response

The effective reduction in circulating inflammatory cytokine levels was demonstrated in in vitro [12] and animal experiments [61], as well as in several clinical studies [51].

Although only the CRP levels were significantly reduced before and after hemoadsorption in our study, there was also a tendency toward a reduction in IL-6 levels, especially in the studies with the highest baseline serum levels of IL-6 [39,46,50].

These results further support the concept of concentration-dependent removal of cytokines, meaning that molecules with higher concentrations are more effectively eliminated than molecules with lower concentrations [62,63]. This also suggests that patients with hyperinflammatory ARDS could potentially benefit the most from HA, which is something worth considering when designing future trials on HA in ARDS. Nevertheless, it is most likely that the sample size was too small, and the scatter of the data was too large to draw firm conclusions for the whole population. It is important to note that CRP due to its large molecular weight is unlikely to be directly adsorbed by adsorbents; hence, its reduction may be an indirect effect, mainly due to the overall attenuation of the inflammatory response. However, CRP reduction during hemoadsorption has also been found and reported by others, which is in line with our findings [64].

One of the consequences of hyperinflammation is the general loss of vasomotor tone reflected by and culminating in hemodynamic instability and vasoplegic shock. Overall, clinical practice and published data suggest that hemoadsorption therapy is frequently accompanied by rapid hemodynamic stabilization indicated by a reduction in vasopressor requirements [65] and an enhanced reduction in plasma lactate concentrations [66]. We were able to pool the data on lactate levels and norepinephrine doses in six studies [14,39,42,43,45,47]. There was a significant reduction in both lactate levels and norepinephrine requirements after hemoadsorption, which is consistent with previous findings [65]. When comparing studies with a control group, although the overall results were non-significant in favor of the HA groups, it is important to emphasize that three out of the four studies reported a significant benefit [46,47,49].

### 4.3. Improvement in Oxygenation

The most important finding of our study is the significant improvement in PaO2/FiO2 ratio after hemoadsorption.

The first RCT on sepsis-induced acute lung injury was published 10 years ago and applied the HA-330 device [49]. Patients treated with hemoadsorption (*n* = 25) versus the controls (*n* = 21) showed a significant removal of plasma and bronchoalveolar lavage TNF-α and IL-1 during the study. There was a significant improvement in PaO2/FiO2, Lung Injury Score, as well as chest X-ray scores on days 3 and 7. Furthermore, the hemoadsorption group showed a significant reduction in the duration of mechanical ventilation, length of intensive care unit stay, and intensive care unit mortality. Most studies included in our systematic review further supported these early findings, as we also found a significant improvement in oxygenation when comparing the values before and after hemoadsorption in our pooled dataset.

Although the explanation for this phenomenon remains mainly hypothetical, some recent in vitro data provide some explanations for these findings. David et al. investigated the putative interplay between circulating cytokines and vascular permeability and found that endothelial integrity was negatively affected in the form of vascular barrier breakdown when treated with serum before cytokine removal, which was prevented when the cells were exposed to serum collected after cytokine removal [33]. In another recent in vitro study, the authors extracted adsorbed proteins from the hemoadsorber after use in patients undergoing on-pump valve surgery for acute endocarditis and applied them to cultured human aortic endothelial cells [67]. Their results indicate, on the one hand, that the removed proteins may exert detrimental effects on the endothelium, and, on the other hand, that their removal by hemoadsorption can potentially prevent endothelial damage via an attenuation of pro-coagulant changes and increased capillary leakage.

As both interstitial edema and blood clot formation could play an important role in ARDS pathophysiology, these in vitro findings may provide the pathophysiological background for the observed significant improvement in oxygenation. Nevertheless, we were unable to pool sufficient studies with control groups to perform a meta-analysis; hence, all of these potentially positive findings require confirmation by future trials.

Finally, in the most severe case of ARDS, when patients require ECMO therapy, a recent summary of the available data suggested that there was a trend toward an effective reduction in inflammatory biomarkers and vasopressor dosage and an improvement in lung function with adjunctive hemoadsorption [68]. The authors also suggested that the combined and early use of extracorporeal membrane oxygenation and hemoadsorption could represent a novel strategy to promote enhanced lung rest in patients with ARDS, a hypothesis that should be tested in the future.

### 4.4. COVID-19

When discussing COVID-19-caused respiratory dysfunction, the different phenotypes of ARDS [69], the presence or absence of hyperinflammation, and the potential effects of adjunctive hemoadsorption in these patients remains a controversial issue and discussing it in detail is beyond the scope of the current review. Nevertheless, almost half of the included articles involved patients with COVID-19-related lung injury, indicating that adjunctive hemoadsorption received special attention during the pandemic and resulted in a large number of publications [70]. In fact, except for a few articles [27,30,32,33,34,36,40,41,47,49] almost all data that we were able to meta-analyze were published on COVID-19 patients [14,15,29,31,35,37,38,39,42,43,44,45,46,48,50,52,55]. Nevertheless, by removing the studies published on non-COVID patients from the analyses, except for PaO2/FiO2, the results did not reach statistical significance anymore, although the tendencies of the results behaved similarly to that of the whole population. It is difficult to explain these findings clearly, but this may be due to the reduced sample size.

### 4.5. Safety and Mortality

None of the included studies reported major treatment-related adverse events. One of the potential side effects of extracorporeal therapies is thrombocytopenia and bleeding complications. The latter was not reported in any of the studies. Regarding thrombocytopenia, we were only able to pool data from three studies reporting platelet counts before and after the treatment [14,39,43]. Although there was a tendency toward decreased platelet count, it did not reach statistical significance and none of the studies reported this as a serious adverse event. It is still uncertain whether thrombocytopenia is related to hemoadsorption per se or whether this is the result of the extracorporeal circulation.

However, one RCT involving 17 COVID-19 patients reported that hemoadsorption was associated with worse 30-day survival (18% vs. 76%) when used during the first days of ECMO support in COVID-19 [50]. It is important to note that the same study showed some baseline imbalances between the two groups, a strikingly low control group mortality, and, in addition, was severely underpowered to show an effect on survival. In our meta-analysis, there was a non-significant difference in mortality between the HA and control patients.

### 4.6. Strengths and Limitations

To the best of our knowledge, this is the first systematic review and meta-analysis on hemoadsorption in ARDS. However, the small number of available studies is a serious limitation of our meta-analysis. It is important to note, that although the estimation of the mean and its standard deviation using the quartiles are widely accepted in the methodology of performing meta-analyses, they only provide estimations of the true values. Therefore, it is important to note that due to the lack of trials with randomization or otherwise reduced confounding, the data we analyzed potentially suffered from skewed distribution, which was also signaled by the fact that most of the included articles used median values in their reporting. As a result of the lack of the randomized, controlled trials, the level of evidence in our findings was low. Furthermore, the number and quality of the studies did not allow us to perform adequately sized pools and analyses on several outcomes, and there may be a publication bias especially in the case reports and smaller case series. “It is important to note that due to the lack of control groups and rigorous prospective design of the included studies, it cannot be excluded that the observed improvement may have resulted from other concomitant treatments as well”.

Another limitation is that we were only able to pool data on the platelet count, but not regarding other safety measures such as bleeding and drug removal. Finally, we cannot comment on long-term effects such as length of hospital stay and length of mechanical ventilation, and the statements regarding mortality must be interpreted with caution.

### 4.7. Implications of This Study

As an implication for further research, further RCTs need to be conducted in order to have higher quality evidence about the effectiveness of HA therapy, and to draw a conclusion for clinicians when and how this therapy should be used in the case of patients with ARDS.

## 5. Conclusions

This is the first systematic review and meta-analysis on the immediate effects of the course of adjunctive hemoadsorption in patients treated with ARDS. Our findings showed positive results regarding the improvement in oxygenation and reduction in inflammatory mediators without causing clinically important device-related adverse events. Our data could be useful for designing high-quality trials in the future to define the exact role of hemoadsorption in ARDS.

## Figures and Tables

**Table 1:** Baseline characteristics of the included articles.

Study, Year	Indication	Patients on HA ^1^ Therapy (*n*)	Control Group (*n*)	Study Design	Cartridge	Average No. of Adsorbents	Timeframe for Use	Results
Acevedo et al., 2021 [38]	ARDS, COVID-19	1	-	Case report	CytoSorb	1	9.2 h	Improved P/F ^2^ ratio, reduction in NA need
Akil et al., 2020 [47]	ARDS, pneumogenic septic shock	13	7	Prospective obs. + post hoc comparison to retrospective historical controls	CytoSorb	min. of 2	each for 24 h	Mortality 0% SG vs. 57% CG, sign, reduction in NA requirement, serum Lactate in SG
Akil et al., 2022 [46]	COVID-19	16	10	Retrospective observational	CytoSorb	Mean of 6 treatments (range 2–21)	24 h each	HA therapy led to hemodynamic stabilization, reduction in inflammation
Alharthy et al., 2020, [39]	ARDS, COVID-19	50	-	Case series	CytoSorb	2 ± 1 vs. 6 ± 2 (survivors vs. non-survivors)	each for 24 h	Improved P/F ratio among survivors, PLT counts were reduced
Berlot et al., 2020 [37]	ARDS, COVID-19	1	-	Case report	CytoSorb	3	each for 24 h	Improved P/F ratio, reduced CRP and IL-6
Berlot et al., 2021 [15]	ARDS, COVID-19	2	-	Retrospective observational	CytoSorb	3	each for 24 h	PaO_2_/FiO_2_ ratio improved or remained stable, CRP, IL-6 decreased after HA
David et al., 2017 [33]	ARDS, influenza pneumonia	1	-	Case report	CytoSorb	1	24 h	Reduction in CRP, PCT, Hgb, and NA requirements and improved P/F ratio 24 h after HA
Geraci et al. 2021 [14]	ARDS, COVID-19	10	-	Case series	CytoSorb	-	2 in the first 24 h, then 1 every 24 h	HA is a feasible and safe treatment, HA reduced inflammatory markers
Huang et al., 2012 [49]	acute lung injury induced by extrapulmonary sepsis	25	21	RCT	HA-330	Once a day for three consecutive days.	each for 2 h	Improving respiratory function, reduction in IL-1 and TNF-a
Huang et al., 2021 [31]	ARDS, COVID-19	1	-	Case report	oXiris	7	each for 24 h	Reduced vasopressor requirements after HA, reduction in inflammatory markers (IL-6)
Kogelmann et al., 2020 [40]	ARDS	7	-	Case series	CytoSorb	4	12–24 h	Improved P/F ratio, decrease in catecholamine need
Kovacevic et al., 2020 [30]	ARDS, H1N1 influenza	1	-	Case report	CytoSorb	2		Decreased vasopressor requirements after HA
La Camera et al., 2019 [32]	ARDS, postoperative patient, pneumonia	1	-	Case report	CytoSorb	2	-	Reduced NA requirement
Lees et al., 2016 [36]	ARDS, PLV+ *S. aureus* pneumonia	1	-	Case report	CytoSorb	1	24 h	Improved oxygenation, decrease vasopressin and NA requirement
Lother et al., 2019 [41]	ARDS, septic shock	3	-	Case series	CytoSorb	mean 1.3 median 1 (1–3)	38.4, 12 and 13.5 h	Reduction in PLT count, no evidence of altered plasmatic coagulation, 67% mortality
Nassiri et al., 2021 [42]	ARDS, COVID-19	26	-	Case series	CytoSorb	mean 2, median of 2 (1–3)	median 35 IQR (18–48)	Significant reduction in inflammatory markers, reduction in NA requirement, improved P/F ratio and SOFA score
Pieri et al., 2022 [48]	ARDS, COVID-19	15	-	Retrospective observational	CytoSorb	3	17 h (mean)	Improved P/F ratio, reduced CRP
Ramírez-Guerro et al., 2020 [29]	ARDS, COVID-19	1	-	Case report	Jafron HA-380	1	10 h	Improved P/F ratio and CT picture
Rampino et al., 2020 [44]	COVID-19	5	4	Case series	CytoSorb	2	4 h sessions in 2 consecutive days	Better clinical course (mortality, pao2/fio2); lymphocyte no. improved; CRP decreased to a greater extent; IL-6, IL-8, and TNF-α decreased; IL-10 remained unchanged
Rieder et al., 2020 [28]	ARDS, COVID-19	1	-	Case report	CytoSorb	1	72 h	Reduction in PLT count, in CRP and IL-6
Rizvi et al., 2020 [35]	ARDS, COVID-19	1	-	Case report	CytoSorb	8	First 4 for 12h, then 4 for 24 h	Reduction in PLT, Hgb, and WBC
Rodeia et al., 2021 [45]	COVID-19	5	-	Case series	CytoSorb	2	Each 24 h	Safe intervention, positive rational behind the therapy, but with low quality evidence
Supady et al., 2021 [50]	COVID-19 pneumonia requiring ECMO	17	17	Rct	CytoSorb	3, 24 h each	72 h	No significant differences for IL-6 were detected between the two groups after 72 h, increased mortality in the Cytosorb group
Träger et al., 2016 [34]	ARDS, postoperative patient	1	-	Case report	CytoSorb	3	85 h in total	Reduction in interleukin 6 and 8, reduction in NA requirements
Wiegele and Krenn et al., 2015 [27]	ARDS, Legionella Pneumonia associated rhabdomyolysis	1	-	Case report	CytoSorb	2	1st: 6 h 2nd: 5 h	Myoglobin levels decreased, reduction in required NA dose, improvement in urinary output
Wunderlich-Sperl et al., 2021 [43]	ARDS, COVID-19	13	-	Case series	CytoSorb	Median of 4 HA treatment (range 1–15 days)	Max of 24 h	Decreased inflammatory parameters after HA, reduction in NA doses, improvement in PF ratio, reduction in PLT count

^1^ HA: hemoadsorption_._^2^ P/F: PaO2/FiO2_._

## Data Availability

The dataset supporting the conclusions of this article is available from the corresponding author.

## Supplementary Materials

The following supporting information can be downloaded at: https://www.mdpi.com/article/10.3390/biomedicines11113068/s1, Figure S1: Pooled data from case reports showing PaO2/FiO2 ratios (mmHg) before and after the treatment (*p* = 0.15); Figure S2: Pooled data from case reports showing CRP levels (mg/L) before and after the treatment (*p* = 0.0078); Figure S3: Pooled data from case reports showing IL-6 levels (pg/mL) before and after the treatment; (*p* = 0.016); Figure S4: Forest plot presenting the difference of mean differences of required norepinephrine (NE) doses in the hemoadsorption (intervention), and standard medical treatment (contol) group before, and after treatment (*p* = 0.106); Figure S5: A—PLT Forest plot of the mean difference of PLT count (G/L) showing a non-significant reduction after hemoadsorption treatment (MD = −57.12 G/L, *p* = 0.186); B—WBC–Pooled data from case reports showing white blood cell count before and after the treatment. (*p* = 0.58); Figure S6: Forest plot of the average length of ICU stay in the experimental (hemoadsoprtion) and in the control group showing no significant differences between the two groups (MD: 1.17 days, *p* = 0.82); Figure S7: Mortality. Forest plot on the risk ratio (RR) of mortality in the experimental (hemoadsoprtion) and in the control group showing no significant differences between the two groups (RR: 0.64, *p* = 0.52); Figure S8: A—Mean difference of PaO2FiO2 (mmHg) after HA treatment, in COVID-19 patients (subgroup “All”); B—Mean difference of serum IL-6 (pg/mL) after HA treatment, in COVID-19 patients (subgroup “All”); 8C—Mean difference of required NE dose (μg/kg/min) after HA treatment, in COVID-19 patients (subgroup “All”); 8D—Mean difference of serum lactate level (mg/L) after HA treatment, in COVID-19 patients (subgroup “All”); Figure S9: A—RCTs – RoB 2 tool; B—Retrospective studies—Robins I; C—Critical Appraisal tools for use in JBI Systematic Reviews—Checklist for case series [71]; D—Critical Appraisal tools for use in JBI Systematic Reviews—checklist for case reports [72]; Table S1: Quality assessment of the included studies (GRADE).
