# Supplementary material for: Hemoadsorption as Adjuvant Therapy in Acute Respiratory Distress Syndrome (ARDS): A Systematic Review and Meta-Analysis
Source: Biomedicines. 2023 Nov 16;11(11):3068. doi: 10.3390/biomedicines11113068 (PMC10669540; doi:10.3390/biomedicines11113068)
Supplement: Supplementary file 1 [file biomedicines-11-03068-s001.zip › biomedicines-2691472-supplementary.pdf]

Hemoadsorption as an adjuvant therapy in acute respiratory distress syndrome (ARDS): a systematic review and meta-analysis

Supplementary material

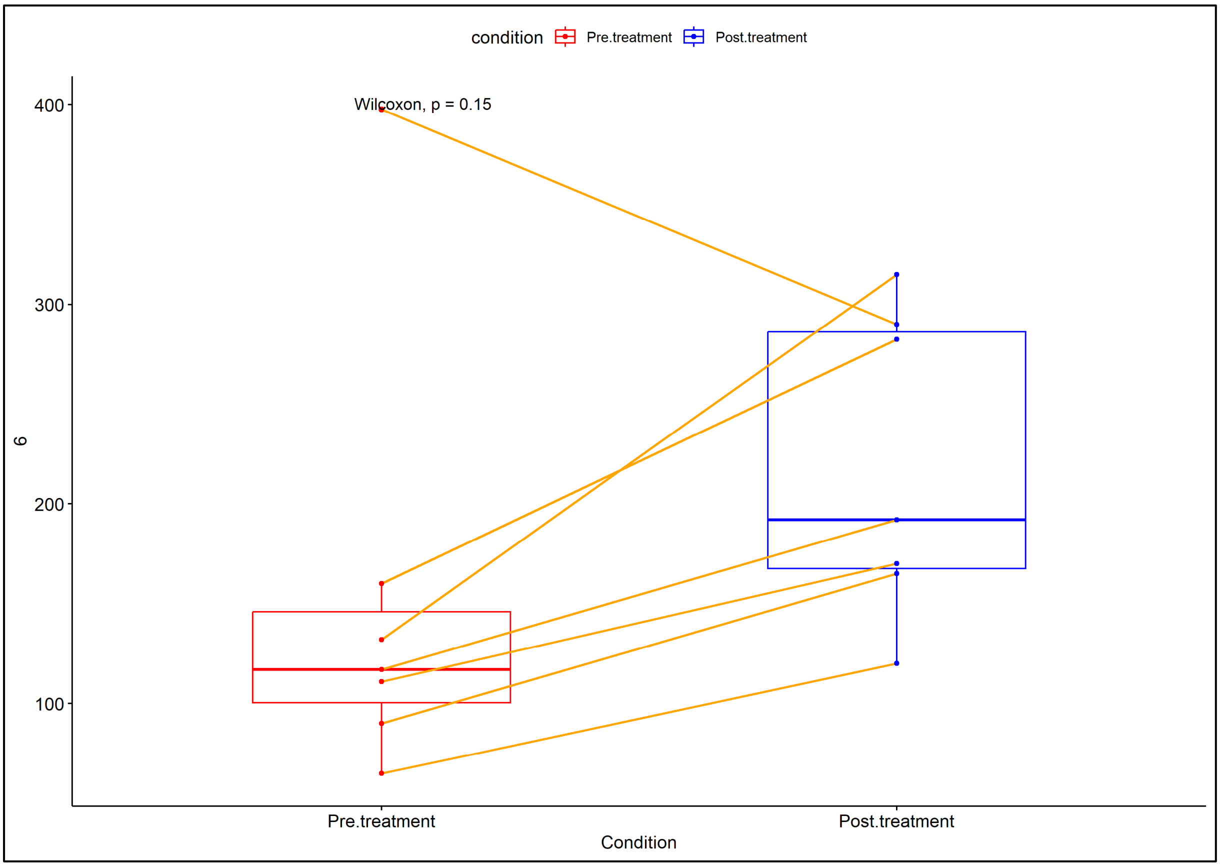

Figure S1. Pooled data from case reports showing PaO_2/FiO_2 ratios (mmHg) before and after the treatment (p=0.15).

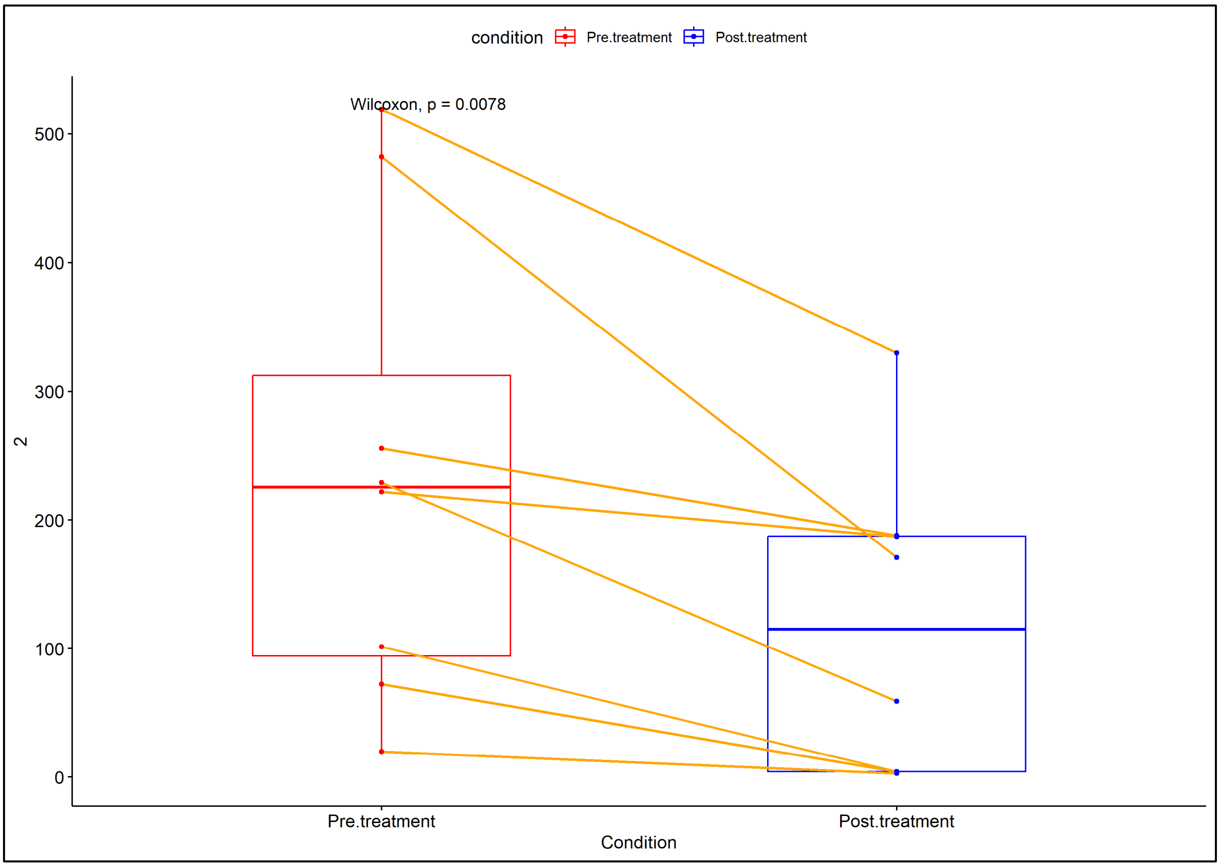

Figure S2. Pooled data from case reports showing CRP levels (mg/L) before and after the treatment (p=0.0078)

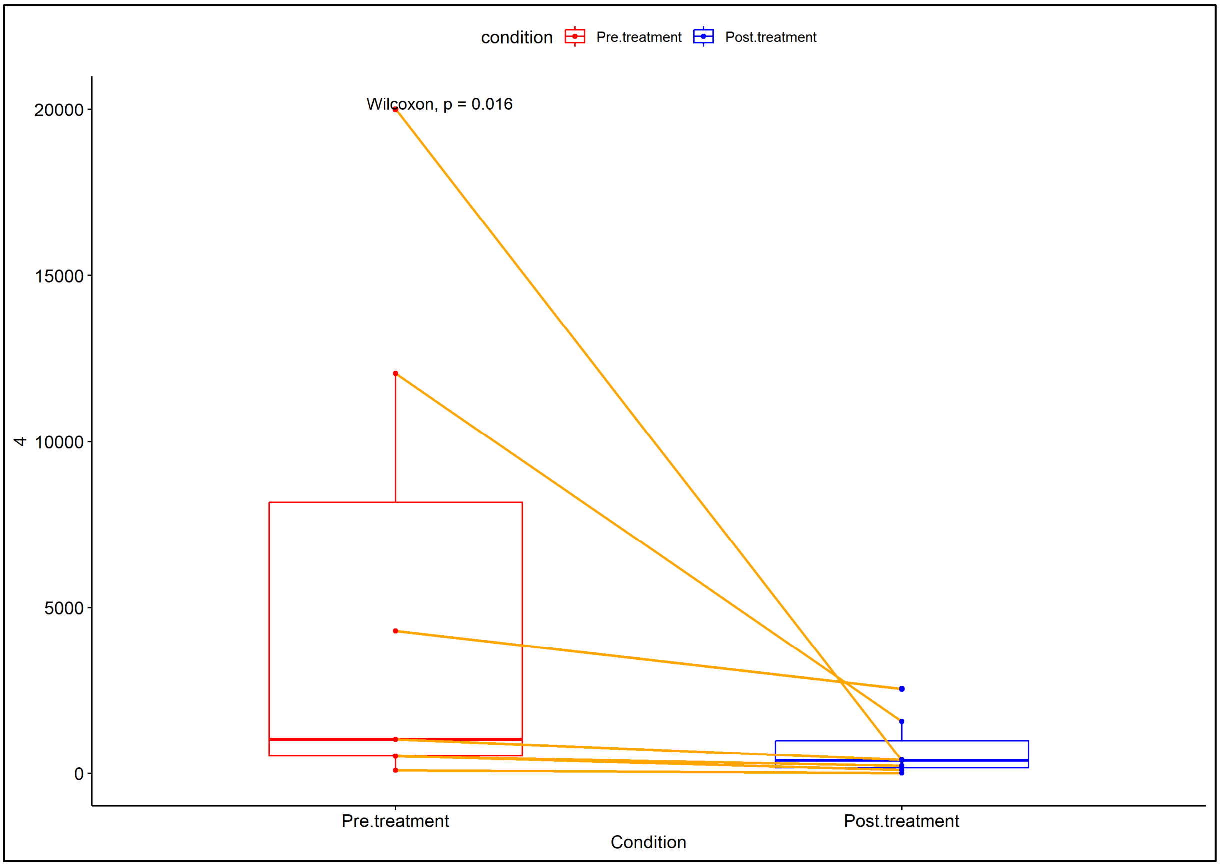

Figure S3. Pooled data from case reports showing IL-6 levels (pg/mL) before and after the treatment. (p=0.016)

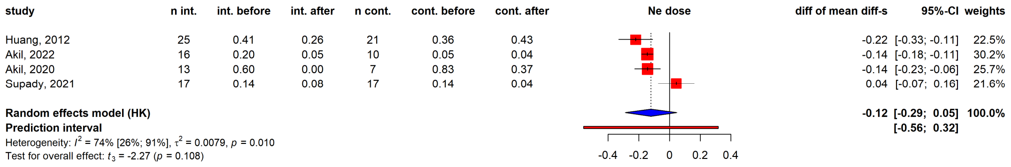

Figure S4. Forest plot presenting the difference of mean differences of required norepinephrine (NE) doses in the hemoadsorption (intervention), and standard medical treatment (contol) group before, and after treatment (p=0.106).

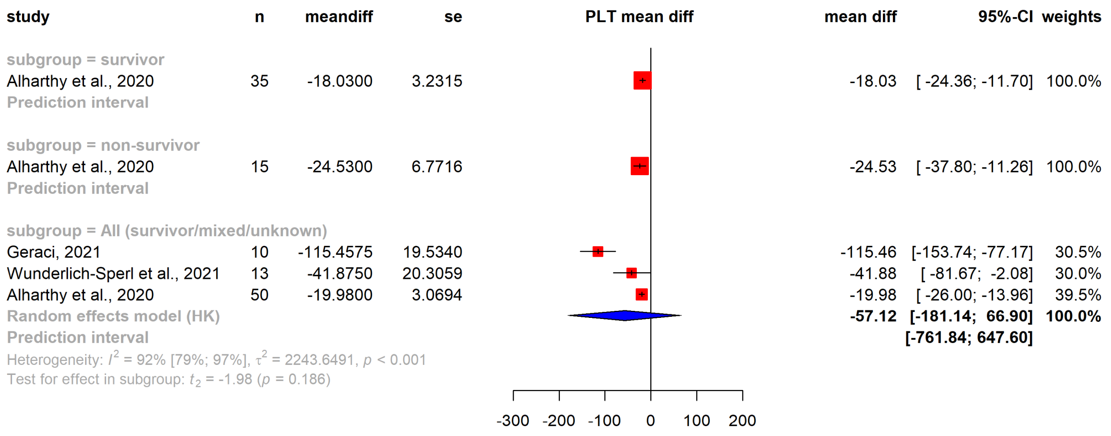

Figue S5A. PLT Forest plot of the mean difference of PLT count (G/L) showing a non-significant reduction after hemoadsorption treatment (MD=-57.12 G/L, p=0.186).

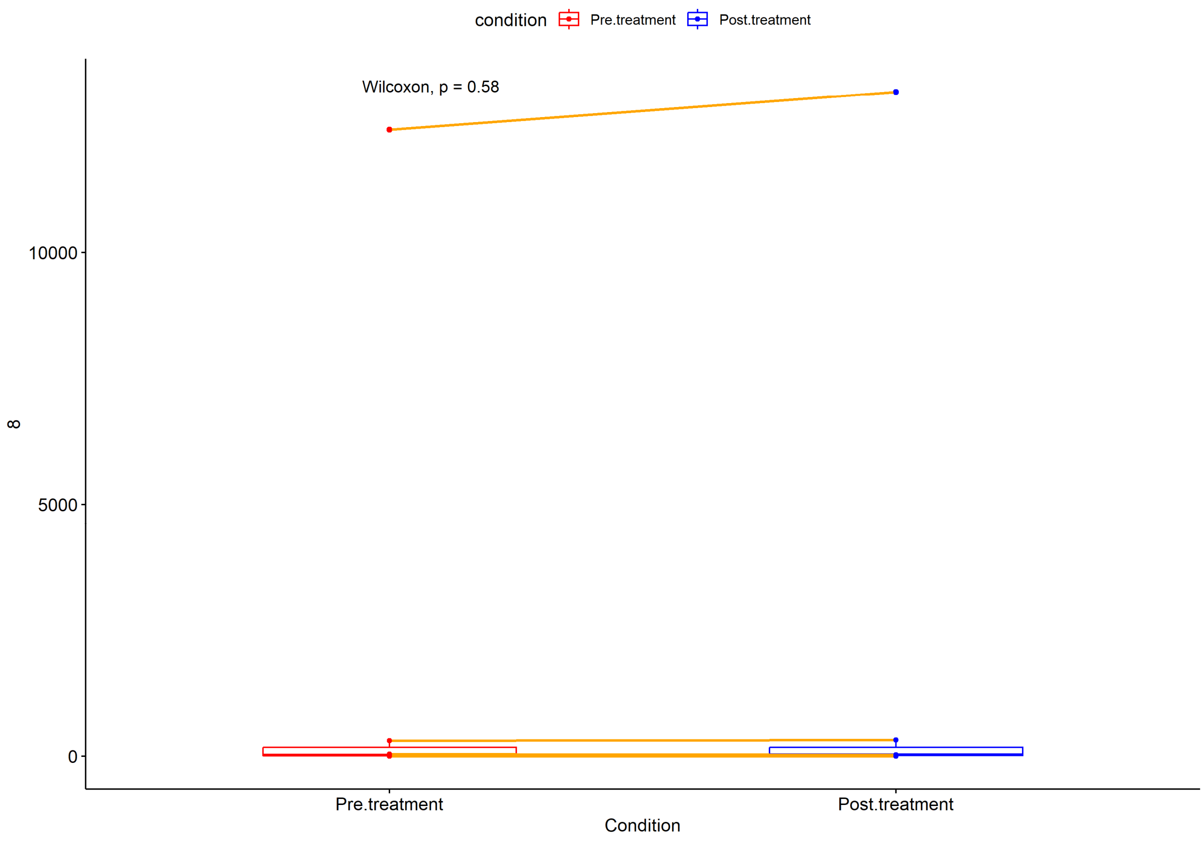

Figure S5B. WBC - Pooled data from case reports showing white blood cell count before and after the treatment. (p=0.58)

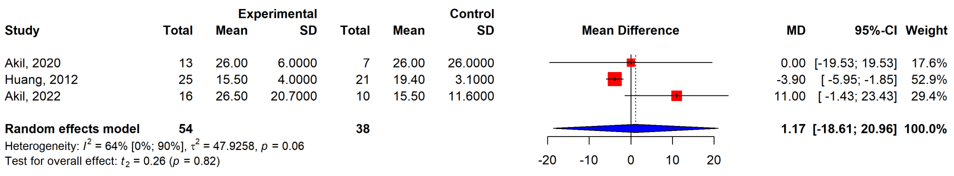

Figure S6. Forest plot of the average length of ICU stay in the experimental (hemoadsoprtion) and in the control group showing no significant differences between the two groups (MD: 1.17 days, p=0.82).

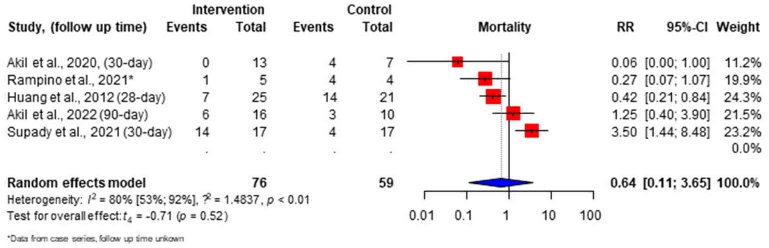

Figure S7. Mortality. Forest plot on the risk ratio (RR) of mortality in the experimental (hemoadsoprtion) and in the control group showing no significant differences between the two groups (RR: 0.64, p=0.52).

COVID-19 subgroup analysis:

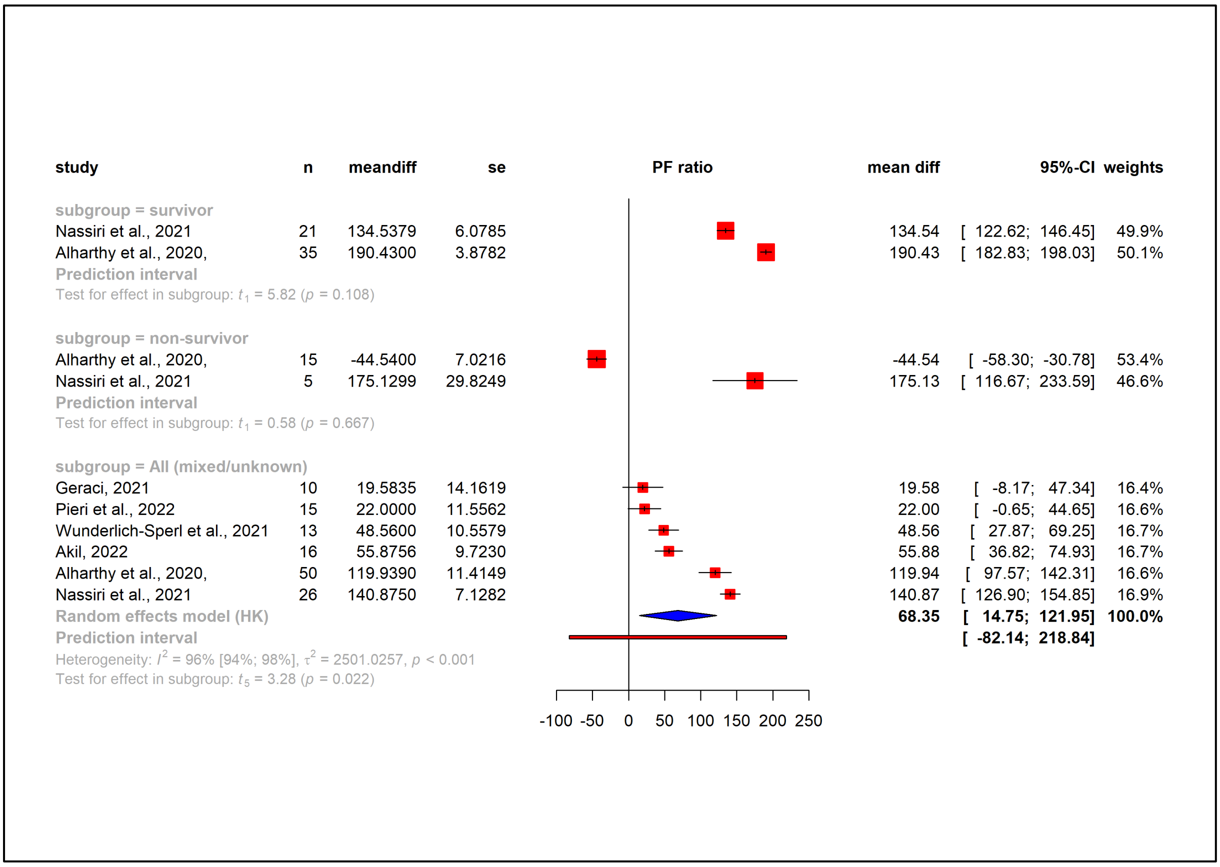

Figure S8A. Mean difference of PaO_2FiO_2 (mmHg) after HA treatment, in COVID-19 patients (subgroup „All”).

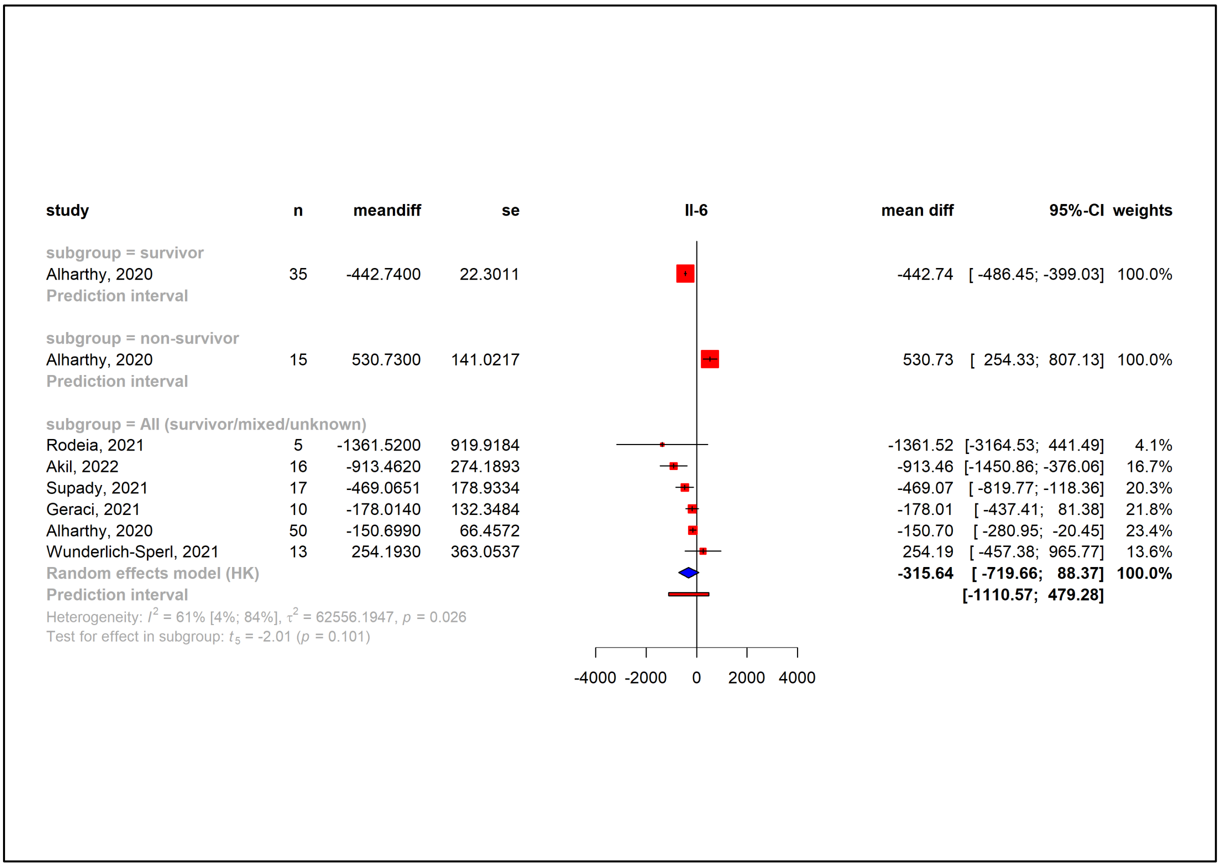

Figure S8B. Mean difference of serum IL-6 (pg/ml) after HA treatment, in COVID-19 patients (subgroup „All”)

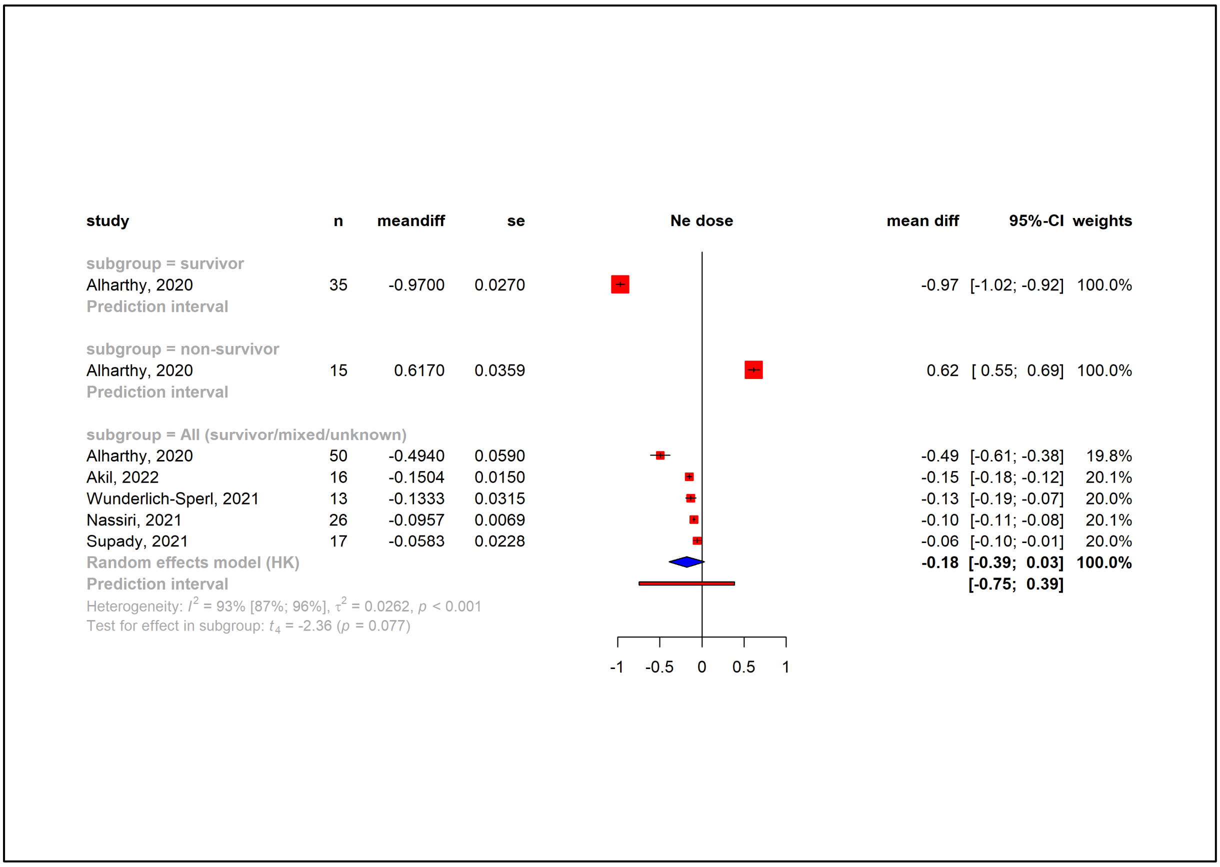

Figure S8C. Mean difference of required NE dose (ug/kg/min) after HA treatment, in COVID-19 patients (subgroup „All”)

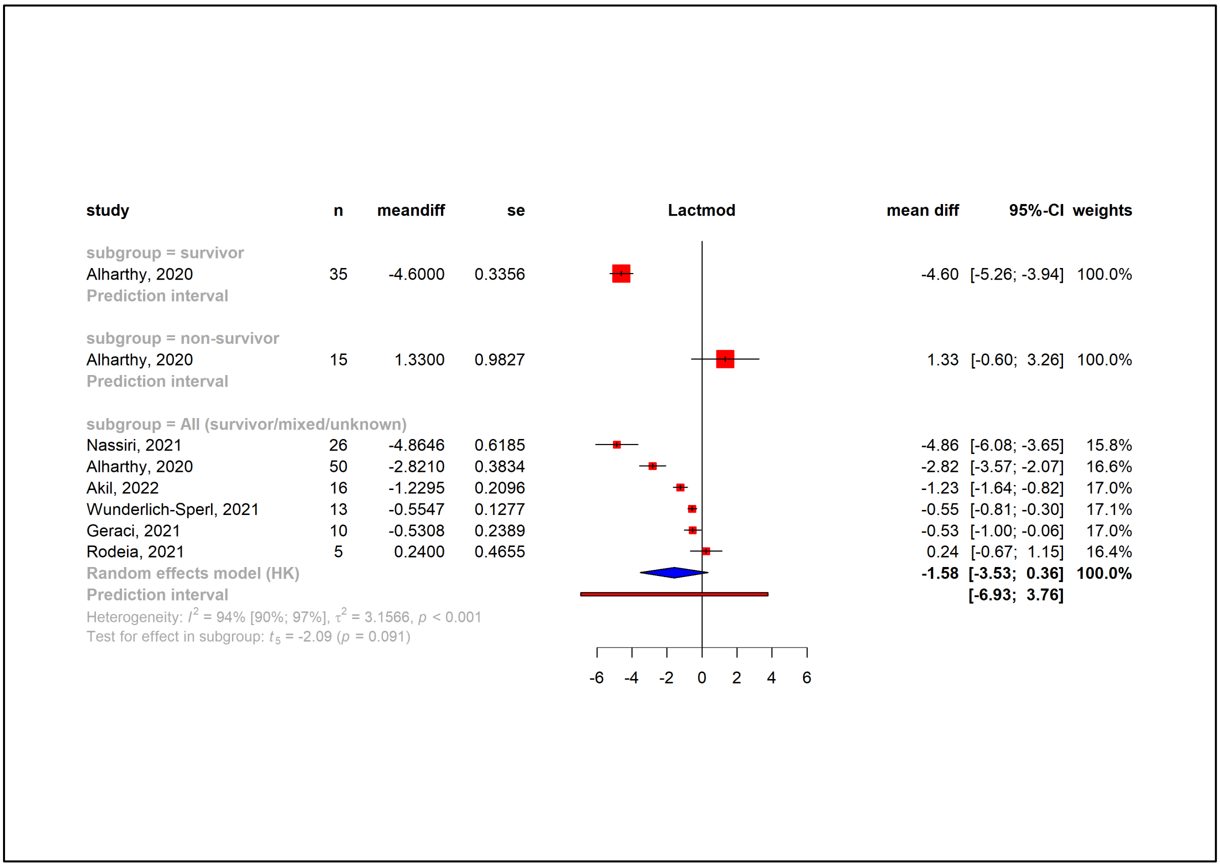

Figure S8D. Mean difference of serum lactate level (mg/l) after HA treatment, in COVID-19 patients (subgroup „All”)

Author(s):

Question: Hemoadsorption (HA) therapy compared to Standard medical treatment for ADS

Setting: critically ill

Bibliography:

Certainty assessment							№ of patients		Effect		Certainty	Importance
№ of studies	Study design	Risk of bias	Inconsistency	Indirectness	Imprecision	Other considerations	Hemoadsorption (HA) therapy	Standard medical treatment	Relative (95% CI)	Absolute (95% CI)		
**Vasopressor support (norepinephrine)**												
4	observational studies	very serious[a]	not serious	not serious	not serious	none	71	55	-	MD **0.12 ug/kg/min lower** (0.29 lower to 0.05 higher)	⨁⨁◯◯ Low	IMPORTANT
**Length of ICU stay**												
3	observational studies	very serious[a]	not serious	not serious	not serious	none	54	38	-	MD **1.17 days higher** (18.61 lower to 20.96 higher)	⨁⨁◯◯ Low	IMPORTANT
**Mortality (follow-up: range 28 days to 90 days)**												
5	observational studies	very serious[a,b]	serious[b]	not serious	not serious	none	28/76 (36.8%)	29/59 (49.2%)	**RR 0.64** (0.11 to 3.65)	**177 fewer per 1 000** (from 437 fewer to 1 000 more)	⨁◯◯◯ Very low	IMPORTANT

CI: confidence interval; **MD:** mean difference; **RR:** risk ratio

Explanations

a. Akil 2021 receives a Critical risk of bias in the domain of "selection of participants into the study". We found no information that the authors have done any matching analysis such as propensity score matching or regression.

b. The outcome in the studies varied from 28 to 90-day mortality

Table S1. Quality assessment of the included studies (GRADE)

RISK OF BIAS ASSESSMENT

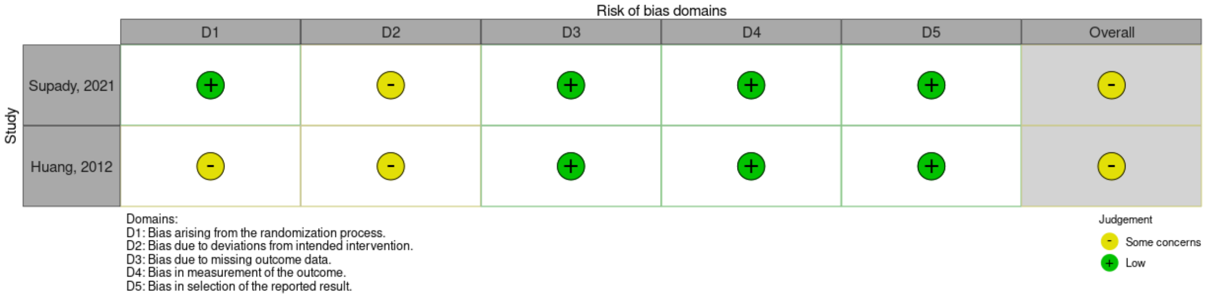

Figure S9A. RCTs – RoB 2 tool

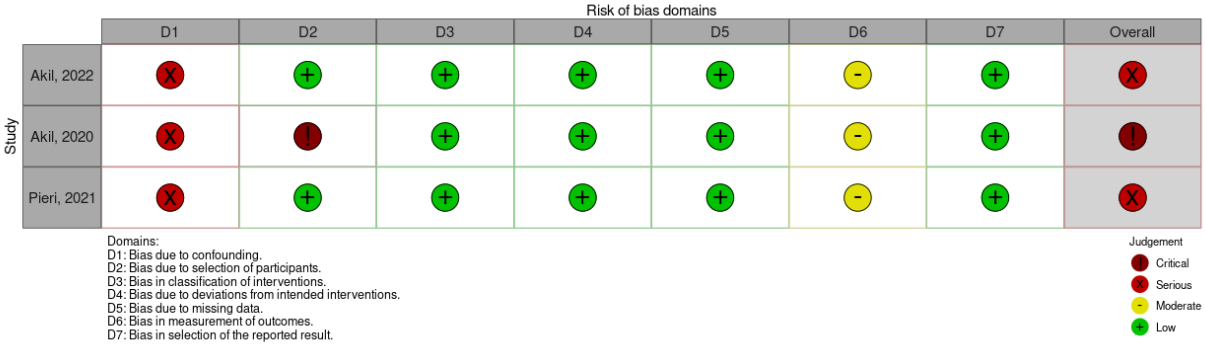

Figure S9B. Retrospective studies – Robins I

Yes / No / Unclear / Not applicable	Alharthy, 2020,	Kogelmann, 2020	Pieri, 2022	Lother, 2019	Nassiri, 2021	Wunderlich-Sperl, 2021	Geraci, 2021	Rodeia, 2021	Rampino, 2021
Were there clear criteria for inclusion in the case series?	Yes	Yes	No	No	Yes	Yes	Yes	Yes	Yes
Was the condition measured in a standard, reliable way for all participants included in the case series?	Yes	Yes	Yes	Yes	Yes	Yes	Yes	Yes	Yes
Were valid methods used for identification of the condition for all participants included in the case series?	Yes	Yes	Yes	Yes	Yes	Yes	Yes	Yes	Yes
Did the case series have consecutive inclusion of participants?	Yes	Yes	Yes	No	Yes	Yes	Unclea	Unclea	Unclea
Did the case series have complete inclusion of participants?	Yes	Unclea	Yes	No	Yes	Yes	No	Unclea	Unclea
Was there clear reporting of the demographics of the participants in the study?	Yes	Yes	Yes	Yes	Yes	Yes	Yes	Yes	Yes
Was there clear reporting of clinical information of the participants?	Yes	Yes	Yes	Yes	Yes	Yes	Yes	Yes	Yes
Were the outcomes or follow up results of cases clearly reported?	Yes	Yes	Yes	Yes	Yes	Yes	Yes	Yes	No
Was there clear reporting of the presenting site(s)/clinic(s) demographic information?	Yes	No	No	No	No	Yes	Yes	No	No
Was statistical analysis appropriate?	Yes	Yes	Yes	Yes	Yes	Yes	Yes	No	No
Overall appraisal	10	8	8	6	9	10	8	6	

Figure S9C. Critical Appraisal tools for use in JBI Systematic Reviews - Checklist for case series (Munn Z, Barker TH, Moola S, Tufanaru C, Stern C, McArthur A, Stephenson M, Aromataris E. Methodological quality of case series studies: an introduction to the JBI critical appraisal tool. JBI Evidence Synthesis. 2020;18(10):2127-2133)

Yes / No / Unclear / Not applicable		Acevedo, 2021	Berlot 2020	Lees, 2016	Rizvi, 2020	Träger, 2016	David, 2017	La Camera, 2019	Huang 2021	Kovacevic, 2020	Ramirez-Guerro, 2020	Rieder, 2020	Wiegele, 2015
1 Were patient's demographic characteristics clearly described?	Participant characteristics clearly defined	Yes	Yes	Yes	Yes	No	Yes	No	Yes	Yes	Yes	Yes	No
2 Was the patient's history clearly described and presented as a timeline?	Clear history presented as a timeline	Yes	No	Yes	Yes	Yes	No	Yes	Yes	Yes	Yes	Yes	No
3 Was the current clinical condition of the patient on presentation clearly described?	Current clinical condition on presentation clearly described	Yes	Yes	Yes	Yes	Yes	Yes	No	Yes	Yes	Yes	Yes	No
4 Were diagnostic tests or assessment methods and the results clearly described?	Diagnostic tests/assessment methods clearly described	Yes	Yes	Yes	Yes	Yes	No	Yes	Yes	No	Yes	Yes	Yes
5 Was the intervention(s) or treatment procedure(s) clearly described?	Intervention clearly described	Yes	Yes	Yes	Yes	Yes	No	No	Yes	No	Yes	Yes	Yes
6 Was the post-intervention clinical condition clearly described?	Post-interventional clinical condition clearly described	Yes	No	Yes	Yes	No	No	No	Yes	Yes	No	Yes	Yes
7 Were adverse events (harms) or unanticipated events identified and described?	Adverse/unanticipated events identified and described	Yes	Unclear	Yes	Yes	Yes	Unclear	Unclear	Unclear	Unclear	Yes	Yes	Unclear
8 Does the case report provide takeaway lessons?	Take-away lessons provided	Yes	Yes	Yes	Yes	Yes	Yes	Yes	Yes	Yes	Yes	Yes	Yes
TOTAL	TOTAL	8	5	8	8	6	3	3	7	5	7	8	4

NKFIHK120706Figure S9 D :Critical Appraisal tools for use in JBI Systematic Reviews – checklist for case reports (Gagnier JJ, Kienle G, Altman DG, Moher D, Sox H, Riley D, CARE Group. The CARE Guidelines: Consensus-Based Clinical Case Reporting Guideline Development. Headache: The Journal of Head and Face Pain, 2013;53(10):1541-1547.).

Search key used for **MEDLINE (via Pubmed), Web of Science, Scopus and CENTRAL**:
(ARDS or "respiratory distress syndrome" or "acute lung injury" or ALI or "hypoxemic respiratory failure") AND (oXiris or Jafron or CytoSorb or hemadsorp* or hemoadsorp* or haemadsorp* or haemoadsorp* or "blood purification" or "cytokine removal")

Searching strategy in **Embase:**
(ARDS or 'respiratory distress syndrome' or 'acute lung injury' or ALI or 'hypoxemic respiratory failure') AND (oXiris or Jafron or CytoSorb or hemadsorp* or hemoadsorp* or haemadsorp* or haemoadsorp* or 'blood purification' OR 'cytokine removal')

Forest plots of the results analyzing the data of COVID-19 populations, regarding the following outcomes:

- **PaO2/FiO2 ratio**
- **Serum IL-6**
- **Required dose of NE**
- **Serum lactate**

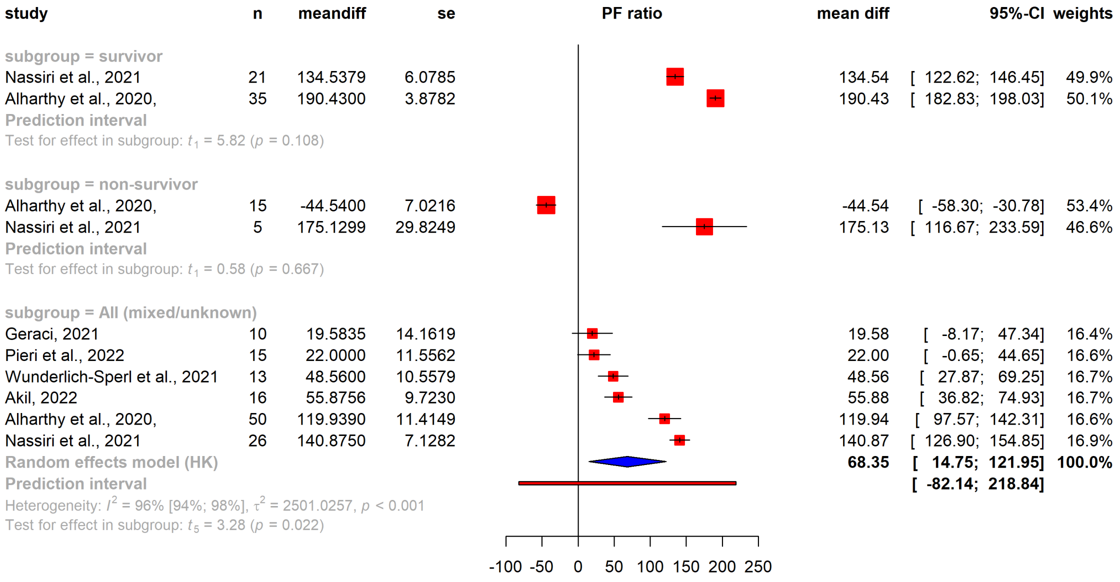

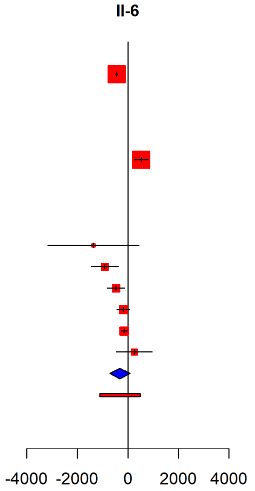

study	n	meandiff	se	mean diff	95%-CI	weights
subgroup = survivor						
Alharthy, 2020	35	-442.7400	22.3011	-442.74	[-486.45; -399.03]	100.0%
Prediction interval						
subgroup = non-survivor						
Alharthy, 2020	15	530.7300	141.0217	530.73	[254.33; 807.13]	100.0%
Prediction interval						
subgroup = All (survivor/mixed/unknown)						
Rodeia, 2021	5	-1361.5200	919.9184	-1361.52	[-3164.53; 441.49]	4.1%
Akil, 2022	16	-913.4620	274.1893	-913.46	[-1450.86; -376.06]	16.7%
Supady, 2021	17	-469.0651	178.9334	-469.07	[-819.77; -118.36]	20.3%
Geraci, 2021	10	-178.0140	132.3484	-178.01	[-437.41; 81.38]	21.8%
Alharthy, 2020	50	-150.6990	66.4572	-150.70	[-280.95; -20.45]	23.4%
Wunderlich-Sperl, 2021	13	254.1930	363.0537	254.19	[-457.38; 965.77]	13.6%
**Random effects model (HK)**				**-315.64**	**[-719.66; 88.37]**	**100.0%**
**Prediction interval**					**[-1110.57; 479.28]**	

Heterogeneity: I^2 = 61% [4%; 84%], τ^2 = 62556.1947, p = 0.026
Test for effect in subgroup: t_5 = -2.01 (p = 0.101)

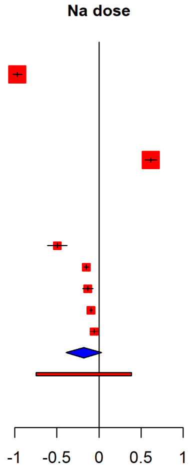

study	n	meandiff	se	mean diff	95%-CI	weights
subgroup = survivor						
Alharthy, 2020	35	-0.9700	0.0270	-0.97	[-1.02; -0.92]	100.0%
Prediction interval						
subgroup = non-survivor						
Alharthy, 2020	15	0.6170	0.0359	0.62	[0.55; 0.69]	100.0%
Prediction interval						
subgroup = All (survivor/mixed/unknown)						
Alharthy, 2020	50	-0.4940	0.0590	-0.49	[-0.61; -0.38]	19.8%
Akil, 2022	16	-0.1504	0.0150	-0.15	[-0.18; -0.12]	20.1%
Wunderlich-Sperl, 2021	13	-0.1333	0.0315	-0.13	[-0.19; -0.07]	20.0%
Nassiri, 2021	26	-0.0957	0.0069	-0.10	[-0.11; -0.08]	20.1%
Supady, 2021	17	-0.0583	0.0228	-0.06	[-0.10; -0.01]	20.0%
**Random effects model (HK)**				**-0.18**	**[-0.39; 0.03]**	**100.0%**
**Prediction interval**					**[-0.75; 0.39]**	

Heterogeneity: I^2 = 93% [87%; 96%], τ^2 = 0.0262, p < 0.001
Test for effect in subgroup: t_4 = -2.36 (p = 0.077)

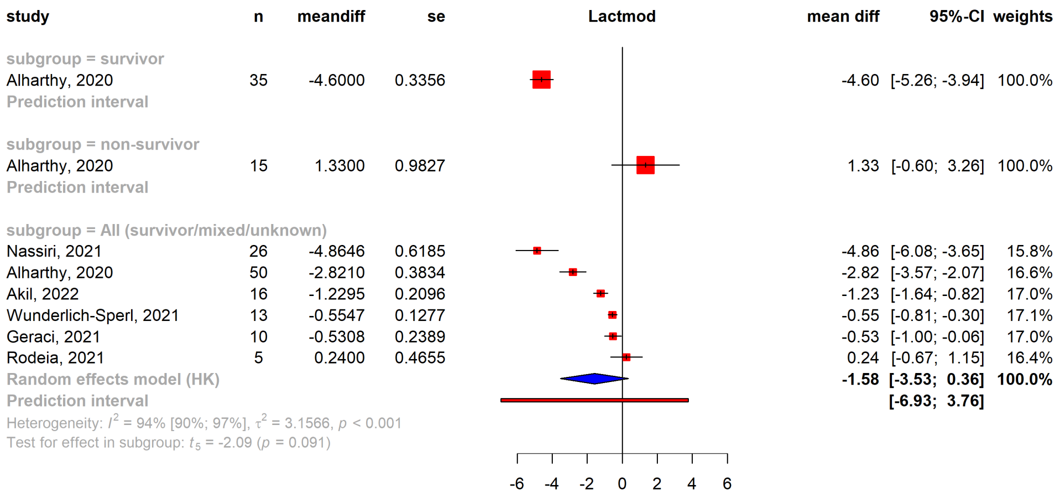
study
n
meandiff
se
Lactmod
mean diff
95%-CI
weights
subgroup = survivor
Alharthy, 2020 35 -4.6000 0.3356 -4.60 [-5.26; -3.94] 100.0%
Prediction interval
subgroup = non-survivor
Alharthy, 2020 15 1.3300 0.9827 1.33 [-0.60; 3.26] 100.0%
Prediction interval
subgroup = All (survivor/mixed/unknown)
Nassiri, 2021 26 -4.8646 0.6185 -4.86 [-6.08; -3.65] 15.8%
Alharthy, 2020 50 -2.8210 0.3834 -2.82 [-3.57; -2.07] 16.6%
Akil, 2022 16 -1.2295 0.2096 -1.23 [-1.64; -0.82] 17.0%
Wunderlich-Sperl, 2021 13 -0.5547 0.1277 -0.55 [-0.81; -0.30] 17.1%
Geraci, 2021 10 -0.5308 0.2389 -0.53 [-1.00; -0.06] 17.0%
Rodeia, 2021 5 0.2400 0.4655 0.24 [-0.67; 1.15] 16.4%
Random effects model (HK) -1.58 [-3.53; 0.36] 100.0%
Prediction interval [-6.93; 3.76]
Heterogeneity: I^2 = 94% [90%; 97%], τ^2 = 3.1566, p < 0.001
Test for effect in subgroup: t_5 = -2.09 (p = 0.091)
-6
-4
-2
0
2
4
6
